# Rheological profile and bioactive compounds content in yogurt incorporated with cherry plum fruit (*Prunus Cerasifera* Ehrh.) powders

**DOI:** 10.3389/fnut.2026.1809167

**Published:** 2026-07-30

**Authors:** Ovidiu Tiţa, Mădălina Moga, Mihaela Adriana Tiţa, Mihai Ognean, Cecilia Georgescu, Adina Frum, Alina Constantina Florea, Maria Adelina Constantinescu

**Affiliations:** 1 Department of Agricultural Sciences and Food Engineering, Lucian Blaga University of Sibiu, Sibiu, Romania; 2 Doctoral School of Lucian Blaga University of Sibiu, Sibiu, Romania; 3 Preclinical Department, Faculty of Medicine, Lucian Blaga University of Sibiu, Sibiu, Romania; 4 National Research and Development Institute for Biotechnology in Horticulture, Stefanesti, Romania

**Keywords:** antioxidants, plant-derived products, polyphenols, *Prunus cerasifera* Ehrh., yogurt

## Abstract

**Introduction:**

The incorporation of plant-based powders into dairy products has attracted increasing attention in recent years due to their ability to enhance the nutritional profile of the final products, making them appealing to consumers seeking healthier options. This study aims to incorporate cherry plum powder into yogurt to improve its nutritional value. Cherry plums represent a valuable ingredient due to their high content of polyphenols, anthocyanins, and antioxidant capacity. Therefore, this study investigates the properties of these powders.

**Methods:**

The effects of powder concentration and storage time on antioxidant activity were evaluated using the DPPH (2,2-diphenyl-1-picrylhydrazyl) method. In addition, rheological and color analyses were performed to assess their influence on the final structure of the yogurt, a key factor for consumer acceptance.

**Results and discussion:**

Textural properties were influenced by storage time. Firmness increased in several samples, reaching up to 169.9 g at day 14, while consistency also showed an increasing trend (up to 4223 g⋅s). Cohesiveness exhibited slight variations, with no consistent trend across samples. Color parameters were only slightly affected by storage, with lightness (L*) remaining relatively stable (76.3–87.8) and minor variations observed in a* and b* values. Total polyphenol content increased markedly with concentration, from 9.2 mg GAE/g at 1% to 19.0 mg GAE/g at 5%. Antioxidant activity showed a slight increase (36.0% to 38.1%), indicating improved functional properties. These findings highlight the potential of cherry plum powder as a functional ingredient in yogurt, contributing to improved nutritional, functional, and rheological properties, and supporting its use in the development of natural dairy products.

## 1 Introduction

The global demand for natural and/or functional products that offer health benefits has exhibited a consistent and sustained growth trajectory, attracting both consumers and manufacturers (1). The adoption of a diet consisting of substantial amounts of fruits, vegetables, and herbs can be attributed to their anti-inflammatory and antioxidant properties (2). Research has demonstrated that the regular consumption of antioxidants can serve as a means of reducing oxidative stress (3) and reduce the risk of osteoporosis, cardiovascular disease (4) and stroke, one of the leading causes of disability worldwide (5). Romania exhibits one of the highest incidence rates of stroke in Europe. This phenomenon is attributed to a combination of factors, including an unhealthy diet deficient in antioxidants, obesity, alcohol and tobacco use, and a sedentary lifestyle (6).

Dairy products, such as milk, yogurt, and cheese, have long been recognized as a fundamental component of a healthy diet (7). Yogurt is a rich source of essential nutrients, including calcium and protein. Numerous studies have demonstrated the therapeutic benefits of yogurt, which is widely regarded as a healthy food due to its high digestibility and efficient nutrient absorption (4). In addition to its health benefits, another key advantage is its versatility. This versatility can be further enhanced by adding ingredients that improve its nutritional value, sensory profile, and texture (8–12).

It has been demonstrated that the addition of agro-industrial byproducts (e.g., citrus pulp, grape pomace, and pulp) to dairy products can enhance their nutritional and physicochemical composition. These byproducts are rich in bioactive compounds, such as polyphenols, which are associated with beneficial effects on human health (3). Enriching dairy products with herbs and natural ingredients aligns with consumer demand for minimally processed foods containing natural extracts. Herbs and spices offer a range of health benefits, including the ability to enhance the taste and aroma of food. In addition, many of these natural products possess antibacterial and anti-inflammatory properties, underscoring their potential health-promoting effects (7).

*Prunus cerasifera* Ehrh. is a widely distributed species in Romania, with a strong capacity to adapt to diverse environmental conditions, including arid environments (13). It is considered a wild tree species, whose fruits have limited economic value. The fruit ripens from early July to mid-September and is characterized by yellow or red coloration (14). In Romania, cherry plums are not subject to industrial processing or distribution through conventional channels, nor are they readily available in food markets (15). However, they are rich in antioxidants and dietary fiber, making them nutritionally valuable. heir consumption remains limited due to their short post-harvest shelf life (16). In other countries, such as France (17–19), Canada (20), and China (21), these fruits are processed (22). The utilization of these resources has the potential to contribute to the development of the local economy and the growth of small businesses. Moreover, organic farming and traditional production methods have the potential to play a significant role in Romania’s efforts to integrate into a circular economy (23–26).

The addition of agri-food byproducts, herbs, and spices to dairy products can enhance their nutritional value, provide therapeutic benefits, and increase consumer appeal (7). de Barros et al. replaced cocoa powder with cacao in dairy beverages and desserts, demonstrating that partial substitution is a viable alternative. This approach enriched the final products with bioactive compounds and increased their antioxidant activity, thereby adding value (27). Arslaner et al. investigated the incorporation of *Hibiscus sabdariffa* L. flower marmalade into yogurt and reported increases in dry matter, ash, titratable acidity, and viscosity, along with decreases in pH, fat, and protein content. The antioxidant properties of the yogurt were significantly improved (28). Similarly, Tang et al. explored the enrichment of yogurt with aqueous extracts from waste cinnamon leaves, reporting positive effects on chemical composition, antioxidant activity, and anti-inflammatory properties, highlighting their potential as a source of functional compounds (29). Corrêa et al. used discarded fruiting bodies of *A. blazei* to obtain an ergosterol-rich extract with strong antioxidant and antibacterial properties. Its incorporation into yogurt significantly enhanced antioxidant capacity without affecting the fatty acid profile or overall nutritional composition (30). Jaster et al. identified strawberry cryoconcentrate as a promising ingredient for improving nutritional value, antioxidant potential, and sensory characteristics (31) Sahingil et al. reported that adding rosehip pulp to yogurt increased total phenolic content and antioxidant capacity, with samples containing 20% rosehip showing the highest consumer acceptance (32). Darwish et al. demonstrated that yogurt fortified with mango or potato peels may help prevent overweight and obesity by reducing weight gain and serum cholesterol levels, while also improving chemical, antioxidant, rheological, and sensory properties (33). Finally, Matar et al. showed that dried apple pulp powder significantly enhances the nutritional, physicochemical, rheological, sensory, and functional properties of low-fat probiotic yogurt, supporting its use as a value-added ingredient (34).

These findings are significant for the processed food industry, as they highlight the potential of natural plant extracts as functional ingredients for dairy products fortification. This strategy is aligns with consumer preferences and emphasizes their potential role in dairy product development (7). This study aims to incorporate cherry plum powder, derived from the whole fruit (including peel and pulp), into yogurt to enhance its nutritional value. Cherry plums are a valuable ingredient due to their high content of polyphenols, anthocyanins, and antioxidant capacity. Accordingly, total anthocyanin content and total polyphenolic compounds were determined for powder. The effect of the powder on the antioxidant activity of the samples was evaluated using the DPPH method. In addition, textural and color analyses were performed. These parameters are essential for understanding the influence of powder concentration on the final structure of the product, a key factor for consumer acceptance. The results of this study may contribute to the development of innovative, functional yogurt products based on cow’s milk, with improved nutritional and textural properties.

## 2 Materials and methods

### 2.1 Sample preparation

**FIGURE 1:**
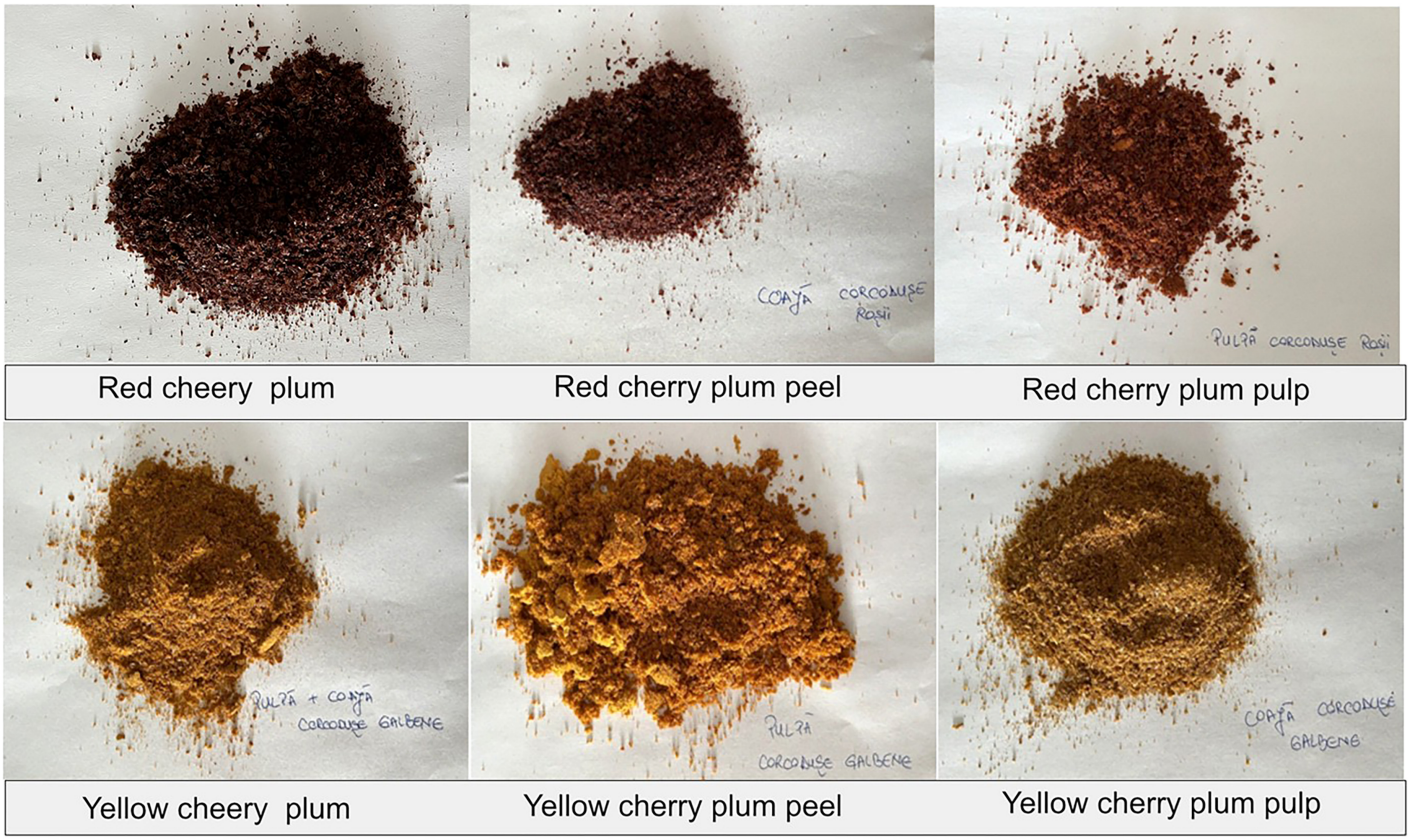
Cherry plums powders.

The red and yellow cherry plums were harvested at physiological maturity. They were washed and separated from their stems and seeds. The fruits were divided into two parts: one was dried whole, while the other was separated into peel and pulp prior to drying. Drying was carried out at 65 °C for approximately 8 h using a fruit and vegetable dehydrator (DD500S, Daewoo, South Korea). This process resulted in a significant reduction in mass. Subsequently, a grinder (TSM6A013B, Bosch, Germany) was used to obtain powders from whole fruits, peels, and pulp (35). The powders were sieved using a mesh size of less than 600 μm. Figure 1 illustrates the cherry plums powders.

The milk was purchased from a dairy producer in Sibiu County, Romania. Once it was confirmed that the milk met the required quality standards, it was pasteurized and used for yogurt production. The powdered cow milk used to produce yogurt has 26% fat and 96% dry matter, and this was purchased from a local store.

The yogurt samples were prepared using 1%, 3%, and 5% (g powder/100 mL yogurt) of red cherry plum (whole fruit, peel, and pulp) and yellow cherry plum (whole fruit, peel, and pulp). The percentages of powder used were established through sensory tests. The maximum acceptable level, based on sensory evaluation, was 5% powder. The results of the sensory analysis were published in a previous article (36).

The yogurt samples were prepared under laboratory conditions, following a recipe developed by the research team that complied with specific technical requirements. The milk was passed through a cotton filter, and then, to destroy pathogenic microorganisms and obtain a microbiologically safe milk, the milk was pasteurized at 85°C for 30 min. After pasteurization, the milk was cooled at a temperature of 45°C and it was inoculated with a bacterial culture containing probiotic strains of Lactobacillus acidophilus La-5 and Bifidobacterium BB-12, Streptococcus thermophilus and Lactobacillus delbrueckii subsp. Bulgaricus (Lacto pro, manufacturer My.yo, Germany). Immediately after inoculation, the milk was mixed with the powdered milk (150 g for 2 L of milk) and then dozed into glass cups with plastic lids along with the cherry plum powder. The mixture was left to ferment for 5 h at a thermostat (manufacturer Electronic April, Romania) at 43°C, during which time bacteria converted lactose into lactic acid, resulting in the coagulation of proteins and the formation of the yogurt texture. After fermentation, the yogurt was cooled to room temperature, then placed in a refrigerator at 4 °C and analyzed on the first, seventh, and fourteenth day (37).

### 2.2 Methods for analyzing milk

The quality of the raw milk was evaluated by determining milk density (aerometric method), titratable acidity (Thörner method), pH (pH-meter), chloride content (Mohr method), the presence of mastitic milk, lactose content (polarimetric method II), fat content (Gerber method, acid-butyrometric method), and protein content (protein titer) (38, 39).

### 2.3 Methods for analyzing cherry plum powder

#### 2.3.1 Determination of dry matter content (DM)

2–5 g of cherry plum powder were weighed with a balance (manufacturer Kern & Sohn, Germany) and placed in a crucible in an oven (UN 110, manufacturer Memmert, Germany) at 105°C for 3 h. They were cooled in a desiccator, and Formula (1) was used to calculate the results (40).


$$\mathrm{DM}\left(\%\right)=\frac{{\text{m}}_{2}-{\text{m}}_{0}}{{\text{m}}_{1}-{\text{m}}_{0}}\cdot 100$$ (1)

where: m_0_ = mass of the empty crucible (g); m_1_ = mass of the crucible with the sample (g); m_2_ = mass of the crucible with the dry sample (g).

#### 2.3.2 Determination of color

A colorimeter (manufacturer HunterLab, USA) was used to determine color. CIELAB parameters were determined, including L* (lightness, ranging from 0 to 100), a* (green-red composite, between -80 and 100), and b* (blue-yellow composite, between -80 and 70) (41).

#### 2.3.3 Extraction of compounds

To determine the total polyphenol content and the antioxidant activity through the 2,2-diphenyl-1-picrylhydrazyl (DPPH) radical scavenging assay, 0.5 g of fruit powder/yogurt was subjected to the extraction process using 10 mL of a solvent composed of methanol and distilled water, in a ratio of 70:30 (v/v). The extraction was carried out in an Erlenmeyer flask equipped with a ground cover, by maintaining it in an ultrasonic water bath (Bandelin Sonorex, manufacturer Bandelin electronic GmbH & Co., KG, Germany) at 40°C, for 30 min and at a frequency of 40 KHz. After cooling, the solution was filtered through a paper filter with a porosity of 8–11 μm into a 10 mL volumetric flask, filling up to the mark with the same solvent (42).

To determine the total anthocyanin content, 0.5 g of each dried fruit powder sample was taken, which was treated with 50 mL of solvent consisting of a mixture of ethanol: 1.5 M hydrochloric acid in a ratio of 85:15 (v/v). The samples were subjected to ultrasonic extraction at 40°C for 30 min at a frequency of 40 KHz, using a water bath (Bandelin Sonorex, manufacturer Bandelin electronic GmbH & Co., KG, Germany), then stored at room temperature, away from direct sunlight, for 24 h. Subsequently, the solutions were filtered through a paper filter with a porosity of 8–11 μm and made up to a final volume of 50 mL in a volumetric flask using the same solvent (42).

#### 2.3.4 Determination of total polyphenol content (TPC)

0.4 mL of hydroalcoholic extract obtained according to the instructions in the previous subsection was measured and added to a test tube. 1 mL of phosphomolybdo-tungstic reagent (Folin-Ciocâlteu reagent), 15 mL of purified water, and 2 mL of 290 g/L sodium carbonate solution were added. The test tube was shaken for 10 min, then placed in a water bath at 40°C for 20 min. After cooling, the absorbance of the sample was measured at 760 nm by using a spectrophotometer (UV-1900, manufacturer Shimadzu, Japan) (42, 43)).

#### 2.3.5 Determination of total anthocyanin content (TAC)

The absorbances of the extracted samples were recorded at λ = 535 nm by using a spectrophotometer (UV 1900, manufacturer Shimadzu, Japan), and the total amounts of anthocyanins, expressed in mg/g dry plant weight (d.w.), were calculated using the Formula (2).


$$\mathrm{TAC}\left(\mathrm{mg}/gd.w.\right)=\frac{E\cdot d}{98.2}$$ (2)

where: E = extinction of the solution; d = dilution factor; 98.2 = absorption value of the solvent ethanol: hydrochloric acid 1.5 M (85:15), at λ = 535 nm (43).

#### 2.3.6 Determination of antioxidant activity - DPPH free radical scavenging assay (RSA)

A stock solution of 25 μg/mL DPPH in methanol was prepared and kept at refrigeration temperature and in the dark for 2 h before use. Then, 970 μL of DPPH stock solution was added to 30 μL of the sample solution. The absorbance was recorded at 515 nm by using a spectrophotometer (UV-1900. manufacturer Shimadzu, Japan), when the value indicated by the spectrophotometer is stable, and the results were expressed as (%) (43–45). The DPPH radical scavenging activity was determined using the following Formula (3).


$$RSA\left(\%\right)=\frac{C_{0}-C_{1}}{C_{0}}\cdot 100$$ (3)

where: C_0_ = concentration of DPPH stock solution (μg/mL), and C_1_ = concentration of DPPH in the sample (μg/mL).

### 2.4 Methods for analyzing yogurt samples

#### 2.4.1 Determination of texture

The back extrusion (pseudo-compression) method was used to determine the texture properties. Back extrusion tests were performed using a Texture Analyzer (TA-XT2i, manufacturer Stable Micro Systems, UK), equipped with a 5 kg load cell. The test parameters were as follows: cylindrical probe diameter (35 mm), test speed [1.0 mm/s (before), 1.0 mm/s (during), 2.0 mm/s (after)], test distance (20 mm), and surface trigger force (10 g). The samples were kept refrigerated, and the texture was measured at a sample temperature of 4°C. The yogurt was homogenized by stirring 20 times with a spoon in a clockwise direction and then transferred to a 125 mL glass container of 64 mm × 70 mm (diameter × height). The textural parameters measured were: maximum positive force in compression (firmness); positive area of the curve, which indicates the internal strength of the bonds in the product (consistency); maximum negative force of the curve, which indicates the force required to withdraw the probe from the sample (cohesiveness) (42, 46).

#### 2.4.2 Determination of color

The method used to determine color is identical to the one described in subsection 2.3.2 (41).

#### 2.4.3 Extraction of compounds. Determination of total polyphenol content. Determination of antioxidant activity

The method used for the extraction of compounds is identical to that described in subsection 2.3.3 (42). The method used to determine the total polyphenol content is identical to that described in subsection 2.3.4 (42, 43). The method used to determine antioxidant activity is identical to that described in subsection 2.3.6 (43–45).

### 2.5 Statistical analysis

Statistical analyses were performed using IBM SPSS 345 Statistics and XLSTAT 2024. The effects of formulation (concentration) and storage time, as well as their interaction, on color, antioxidant, and textural parameters were evaluated using two-way analysis of variance (ANOVA), followed by Duncan’s multiple range test at a significance level of *p* < 0.05.

Additionally, to better understand the relationships among the parameters that characterize yogurt, data regarding its physicochemical analysis (36) were included in the statistical processing using Pearson correlation analysis and hierarchical cluster analysis (HCA). Pearson correlation analysis was performed using IBM SPSS Statistics to assess the relationships between physicochemical (acidity, water holding capacity, syneresis), antioxidant (total polyphenols, DPPH), color (L*, a*, b*), and textural parameters (firmness, consistency, cohesiveness). The strength and direction of the correlations were expressed by the Pearson correlation coefficient (r), and statistical significance was evaluated at *p* < 0.05 and *p* < 0.01. Hierarchical cluster analysis (HCA) was performed using XLSTAT 2024, based on Ward’s method and Euclidean distance, to evaluate similarities among samples and to identify grouping patterns.

## 3 Results and discussion

### 3.1 Physicochemical analysis of raw milk

Table 1 presents the results of the physicochemical analyses performed on raw cow’s milk. The acidity of the milk is 16.06 °T and the pH is 6.6. The chloride content is 0.426%, the lactose content is 2.94%, the fat content is 3.7%, and the protein content is 3.4 g/100 mL. The results obtained fall within normal limits and confirm that the milk can be used to make yogurt.

**TABLE 1:** Milk quality assessment results (mean ± standard deviation).

Evaluated parameter	Results
Density, g/cm^3^	1.032 ± 0.001
Titratable acidity, T	16.06 ± 0.12
pH	6.6 ± 0.05
Chloride content, %	0.426 ± 0.001
Mastitis milk	negative
Lactose content, %	2.94 ± 0.03
Fat content, % Protein content, g/100 mL	3.7 ± 0.1 3.4 ± 0.1

The results are expressed as mean ± standard deviation (SD), *n* = 3.

### 3.2 Analysis of cherry plum powders

#### 3.2.1 Determination of dry matter content and color

As illustrated in Table 2, the results obtained from the determination of the dry matter content and color of cherry plum powder are presented. The dry matter content ranges from 89.92% (red cherry plum powder) to 92.84% (red cherry plum pulp). In the case of yellow cherry plum, the highest dry matter content is found in the pulp powder (91.87%), and the lowest in the peel powder (91.49%). It has been demonstrated that powders with a low moisture content are less susceptible to microbiological degradation over time and exhibit greater stability (47). Săpoi et al. also reported a moisture content of 82.65% (48). In terms of color, the lightness (L* values) is higher in yellow cherry plum powder. This type of powder has values ranging from 36.20 to 41.18, compared to red cherry plum powder, which has values ranging from 19.92 to 34.93. The redness (a* values) decreased significantly in the case of yellow cherry plum (13.18–16.89), while the values for red cherry plum range from 15.04 to 18.78. Similar results were also obtained by Săpoi et al., whose cherry plum samples had a lightness of 39.49 and a redness of 17.06 (48). This phenomenon can be attributed to the divergent reactions of the pigments when exposed to elevated temperatures. It has been demonstrated that upon desiccation, the red pigments present in red cherry plum transform a brown hue, while the yellow pigments present in yellow cherry plum undergo a darkening (49).

**TABLE 2:** Cherry plum powder analysis (mean ± standard deviation).

Sample name	Dry matter content %	Color		
		L*	a*	b*
Red cherry plum powder	89.92 ± 0.005^e^	19.92 ± 0.01^f^	15.04 ± 0.005^d^	9.52 ± 0.01^f^
Red cherry plum peel powder	91.64 ± 0.005^c^	26.74 ± 0.01^e^	17.61 ± 0.02^b^	11.49 ± 0.02^e^
Red cherry plum pulp powder	92.84 ± 0.02^a^	34.93 ± 0.09^d^	18.78 ± 0.005^a^	29.60 ± 0.10^c^
Yellow cherry plum powder	91.65 ± 0.01^c^	40.14 ± 0.005^b^	16.89 ± 0.005^c^	31.78 ± 0.01^a^
Yellow cherry plum peel powder	91.49 ± 0.01^d^	41.18 ± 0.005^a^	13.18 ± 0.005^e^	27.79 ± 0.01^d^
Yellow cherry plum pulp powder	91.87 ± 0.005^b^	36.20 ± 0.02^c^	16.83 ± 0.05^c^	30.19 ± 0.01^b^

The results are expressed as mean ± standard deviation (SD), *n* = 3. The results in the same column followed by the same lowercase letters are not significantly different (*p* > 0.05).

#### 3.2.2 Determination of total polyphenol content, anthocyanin content, and antioxidant activity

As illustrated in Table 3, the results demonstrate the total polyphenol content, anthocyanin content, and antioxidant activity in cherry plum powder.

**TABLE 3:** Total polyphenols content, total anthocyanins content, and antioxidant activity in cherry plum powder samples (mean ± standard deviation).

Sample name	TPC mg GAE/g d.w.	TAC mg/g d.w.	RSA%
Red cherry plum powder	3.24 ± 0.08^a^	1.11 ± 0.13^b^	35.02 ± 0.36^a^
Red cherry plum peel powder	3.60 ± 0.41^a^	1.71 ± 0.04^a^	31.61 ± 0.67^b^
Red cherry plum pulp powder	1.77 ± 0.20^b^	0.63 ± 0.05^c^	23.24 ± 0.06^c^
Yellow cherry plum powder	2.87 ± 0.13^a^	0.55 ± 0.05^c^	16.3 ± 0.48^e^
Yellow cherry plum peel powder	3.24 ± 0.19^a^	0.56 ± 0.05^c^	17.24 ± 0.73^e^
Yellow cherry plum pulp powder	3.71 ± 0.04^a^	0.52 ± 0.02^c^	20.57 ± 0.18^d^

TPC, total polyphenol content; TAC, total anthocyanin content; RSA, radical scavenging assay (antioxidant activity). The results are expressed as mean ± standard deviation (SD), *n* = 3. The results in the same column followed by the same lowercase letters are not significantly different (*p* > 0.05).

Yellow cherry pulp has a polyphenol content of 3.71 mg GAE/g d.w., red cherry peel powder has a content of 3.60 mg GAE/g d.w., and red cherry pulp powder has a content of 1.77 mg GAE/g d.w. In the case of red cherry plums, the peel has the highest amount of polyphenols compared to the other red cherry plum samples. In the case of yellow cherry plums, the pulp has the highest amount of polyphenols compared to the other yellow cherry plum samples. In the study conducted by Wang et al., an analysis was performed on the fruit and peel of cherry plums. The findings revealed that the total phenolic content ranged from 1.34 to 6.11 g GAE/kg of fresh weight (50). Consistent with our results, Ternjak et al. (51) found values of 277 mg GAE/100 g for yellow cherry plum, while Săpoi et al. (48) reported an average TPC content of 135.64 mg GAE/100 g in the peel of cherry plum. Lower values were obtained by Sottile et al. (52), ranging from 34.52 to 97.63 mg GAE/100 g for cherry plum pulp. Due to their significant polyphenol content, several studies have highlighted the importance of using cherry plums in the production of anti-inflammatory drugs (53, 54).

It has been demonstrated that the level of anthocyanin in red plums exceeds that of yellow plums. Red plum peel powder has been found to contain 1.71 mg/g d.w. of total phenolics, with yellow plum peel powder containing 0.55 mg/g d.w. This phenomenon can be attributed to the presence of these pigments, which are responsible for imparting the characteristic red/purple hue to the fruit. The presence of anthocyanins has been demonstrated to contribute, at least in part, to the antioxidant activity of the samples. Drying can affect anthocyanin stability, as these pigments are sensitive to heat, oxygen, and pH. High temperatures and prolonged drying can lead to partial degradation and loss of color (55). Consistent with our results, Sottile et al. (52) reported values ranging from 20.24 to 55.33 cyanidin-3-O-glucoside per 100 g.

The antioxidant activity of red plum powder was recorded at 35.02%, while that of red plum peel powder was recorded at 31.61% and that of red plum pulp powder was 23.24%. With regard to the values obtained for yellow plum powders, the antioxidant activity for yellow plum pulp is 20.57%, and for yellow plum powder it is 16.30%. A study conducted by Gündüz et al. found that the antioxidant activity of 18 selected cherry plum varieties ranges from 0.123 to 0.481 mmol TE/kg fresh weight (FRAP method) and from 0.127 to 0.420 mmol TE/kg fresh weight (TEAC method) (53). Săpoi et al. (48) report a low RSA value of 0.53 mmol Trolox/100 g. Other previous studies have reported a similar trend in the antioxidant content of cherry plums (pulp and fruit) (52, 56).

### 3.2 Yogurt sample analysis

#### 3.2.1 Textural, color, TPC and RSA characteristics of yogurt samples

The textural, color, TPC and RSA characteristics of the analyzed yogurt samples are presented in Supplementary Table 4, as mean values ± standard deviation (*n* = 3). The results highlight the influence of both formulation (concentration) and storage time on the evaluated parameters.

Textural properties were influenced by storage time. Firmness increased in several samples, reaching values up to 169.9 g at day 14, while consistency also showed an increasing trend (up to 4223 g⋅s). Cohesiveness exhibited slight variations, without a consistent trend across all samples. Also, other authors (57–61) associate the increase in firmness with greater stability and density in the samples, as indicated by a more compact texture and improved consistency. A study undertaken by Hashim et al. (11) sought to ascertain the impact of incorporating diverse hydrolysates on the texture and functional characteristics of yogurts. The study demonstrated that the firmness and texture of the analysed samples exhibited comparable trends in terms of sample variation during storage. A parallel tendency in sample firmness to that of sample consistency was also observed in a study conducted by Li et al. regarding the development of a symbiotic yogurt with *Lacticaseibacillus paracasei* and lactitol (62). Cohesion is defined as the degree of deformability of the material before it breaks, and is related to the material’s internal strength (46). Evaluating yogurt with the addition of pineapple pomace, some researchers (58) observed that when the amount of pineapple pomace in the yogurt was increased, the textural parameters increased, but they reported a substantial decrease in the cohesion of the yogurts during the 15-day storage period. This decline in cohesion resulted in products that exhibited a smoother structure. A comparable outcome of diminished cohesion was witnessed upon the incorporation of grape pomace into yogurt (63). It has been demonstrated that the lack of cohesion in yogurt is indicative of a smoother structure (58) According to Wang and his collaborators, powdered apple pomace obtained by freeze-drying can be added to increase the stability of yogurt texture during storage. This effect could be attributed to the gelling capacity of pectins and other soluble fibers released from apple pomace into milk, as well as the strengthening of the gel structure by insoluble particles in apple pomace (64).

Color parameters were slightly affected by storage time. Lightness (L*) remained relatively stable, with values between 76.3 and 87.8, while a* and b* values showed minor variations, indicating limited changes in color during storage. The stability of the pigment is influenced by the method of processing the vegetable before use, and by temperature over time (65). Other authors (66) observed during the storage period of yogurt colored with an anthocyanin-rich pigment prepared from Jabuticaba (*Myrciaria cauliflora* Mart.) peel that L* varied across all concentration levels; however, although these variations were considered statistically significant (*p* < 0.05), they emphasize that the behavior was similar for all samples and slight; therefore, from a technological standpoint, it should not affect visual perception, while the a* color decreased during storage, particularly for higher concentration levels, which could indicate anthocyanin degradation, and b* values changed significantly during storage, but without a clear trend. In yogurts, color plays an important role in consumer acceptance of the product. A study (67) shows that there were differences in the rate of pigment degradation between yogurts made from different fruit species. The half-life of pigments in yogurt mixed with strawberry, cherry, and blueberry preparations was found to be 5.5, 6.7, and 19.0 weeks.

Total polyphenol content showed variable behavior during storage. While some samples exhibited higher values at intermediate storage (day 7), a decrease was observed at day 14 in certain cases (e.g., from 12.489 to 7.401 mg GAE/g for 1% samples), suggesting possible degradation of bioactive compounds. Antioxidant activity (DPPH) showed moderate fluctuations, with values ranging between 35.8% and 39.0% across storage time. Other authors show that the levels of polyphenols increased during storage, presumably due to the gradual release of bound phenolics from mulberry pomace-protein or mulberry pomace-polysaccharide complexes. This finding lends further support to the hypothesis that bound polyphenols are released gradually and are not readily extracted by aqueous-organic systems (26, 68–70). Other authors also observed a degradation of antioxidants in yogurt with the addition of by-products over time, and they associate that with the decomposition of phenolics by polyphenol oxidase (71, 72). Compounds with antioxidant activity are sensitive to light, heat, and acidic environments. Also, various antioxidant activities may vary depending on the solvent used for extraction and the method (73). In yogurts enhanced with mango and banana peel, a slight decrease in polyphenol content and antioxidant activity was observed over time (74). Overall, storage time influenced fermentation dynamics, structural stability, and textural properties of yogurt samples, with more pronounced effects observed after 14 days.

#### 3.2.2 Effect of formulation (concentration)

The influence of formulation (concentration) on the color, antioxidant, polyphenols and textural properties of yogurt samples is presented in Supplementary Table 5.

Textural parameters showed notable changes with concentration. Firmness increased from 146.6 g at 1% to 150.0 g at 5%, while consistency increased from 3722 to 3863 g⋅s, indicating a stronger gel network at higher concentrations. Cohesiveness showed slight variations, with values ranging between 152.9 and 175.2. The texture of yogurts, such as firmness, consistency, and cohesiveness can be improved by adding functional powder ingredients. The structural arrangement of proteins and the microstructure of the protein network have been demonstrated to affect the rheological and textural characteristics of fermented dairy products (75). An enhancement in the evaluated textural parameters was also observed when pumpkin peel, blueberry pomace, and carrot cell walls were added to yogurt, due to the fiber’s properties to reduce syneresis, increase viscosity, and improve yogurt texture (75–79). In addition, a further study proposed by Meena et al. demonstrated that the incorporation of pineapple pomace powder (0.1, 0.25, 0.5%) exerted a significant influence on the physical characteristics of yogurts. Specifically, the addition of pineapple pomace powder resulted in an enhancement of firmness and consistency, although the growth rate of consistency was comparatively weaker (58). Wang et al. (64) also highlighted an improvement in yogurt texture in a dose-dependent manner when apple pomace was added in powdered form to yogurt.

Color parameters were significantly influenced by formulation. L* values decreased from 87.4 to 76.3, indicating a reduction in lightness, while a* values increased from 1.8 to 5.7 and b* values from 12.6 to 15.5, reflecting increased color intensity. The same trend of decreasing L* values and increasing a* values, accompanied by a decrease in b* (given that bilberry pigments are predominantly red), along with an increase in the amount of added powder, has also been reported by other authors who used bilberry pomace powder in proportions of 0.5%, 1.0%, and 1.5% (w/w) in stirred yogurt (47). Similarly, increasing the powder content in yogurt fortified with lemon peel led to a decrease in the L* value, and an increase in a* and especially b*, indicating the predominance of a yellowish hue in the product (80). Other authors have also observed a decrease in lightness and an increase in the a* and b* parameters, depending on the type of pigments contained in the ingredient used to fortify the yogurt, as its concentration in the product increases (66, 74, 81, 82).

Total polyphenol content showed a marked increase with concentration, from 9.2 mg GAE/g at 1% to 19.0 mg GAE/g at 5%. Antioxidant activity (DPPH) also exhibited a slight increase, from 36.0% to 38.1%, suggesting improved functional properties. It has been demonstrated in other studies that the addition of mulberry pomace to yogurt results in an increase in the total polyphenol content, in a dose-dependent manner, from 0.70 to 3.92 mg GAE/g product (46, 68–70). Another study conducted by Frumento et al. in 2013 investigated the effects of adding grape pomace from different varieties to fortify yogurt, developing an acidic lactic product. The study demonstrated that the total polyphenol content could vary from 18.5 to 48.9 mg GAE/g, depending on the grape variety from which the pomace resulted (83). However, adding natural functional ingredients to yogurts significantly increased the antioxidant activity of yogurts, and this effect is attributed to the high nutritional value of natural functional ingredients such as polyphenols, polysaccharides, amino acids, vitamins, and others (84). Overall, increasing concentration improved structural stability, enhanced antioxidant content, contributing to improved overall quality of yogurt samples. Significant differences (*p* < 0.05) were observed between concentrations for most parameters.

#### 3.2.3 Analysis of the relationships between variables using Pearson’s correlation and Hierarchical cluster analysis

Given the significance and impact of yogurt’s physicochemical parameters—including titratable acidity, whey separation (draining and centrifugation), and water holding capacity—on the product’s textural stability and shelf life, it was deemed essential to incorporate these data into the statistical analysis to obtain a more comprehensive and integrated perspective. Consequently, the data processed in a preceding study (36) concerning the acidity of plum yogurt, syneresis by centrifugation and drainage, and water holding capacity were incorporated into this study. The objective was to ascertain the relationship between the physicochemical and textural parameters of the yogurt, as well as to determine the similarity among samples based on all analyzed parameters.

As demonstrated in previous studies (85) water holding capacity is among the most significant physical properties contributing to the stability of yogurt texture. This property reflects the ability of yogurt to retain its own water or water added during processing, such as centrifugation, heating, or pressure, against the forces of gravity. Several authors have proposed a potential association between polyphenols and yogurt proteins, suggesting that this interaction may result in alterations to protein structure, leading to enhanced protein affinity. This, in turn, could promote protein cohesion, augment water holding capacity, and mitigate the occurrence of syneresis in yogurt (84, 86–89). Furthermore, the pH and acidity of yogurt directly influence the product’s color and thus its acceptability, as well as the availability of bioactive compounds incorporated into the product during yogurt fortification with fruit (67).

##### 3.2.3.1 Pearson correlation analysis

Pearson correlation analysis was performed to evaluate the relationships between physicochemical and textural parameters of yogurt samples. The correlation coefficients are presented in Supplementary Table 6.

**FIGURE 2:**
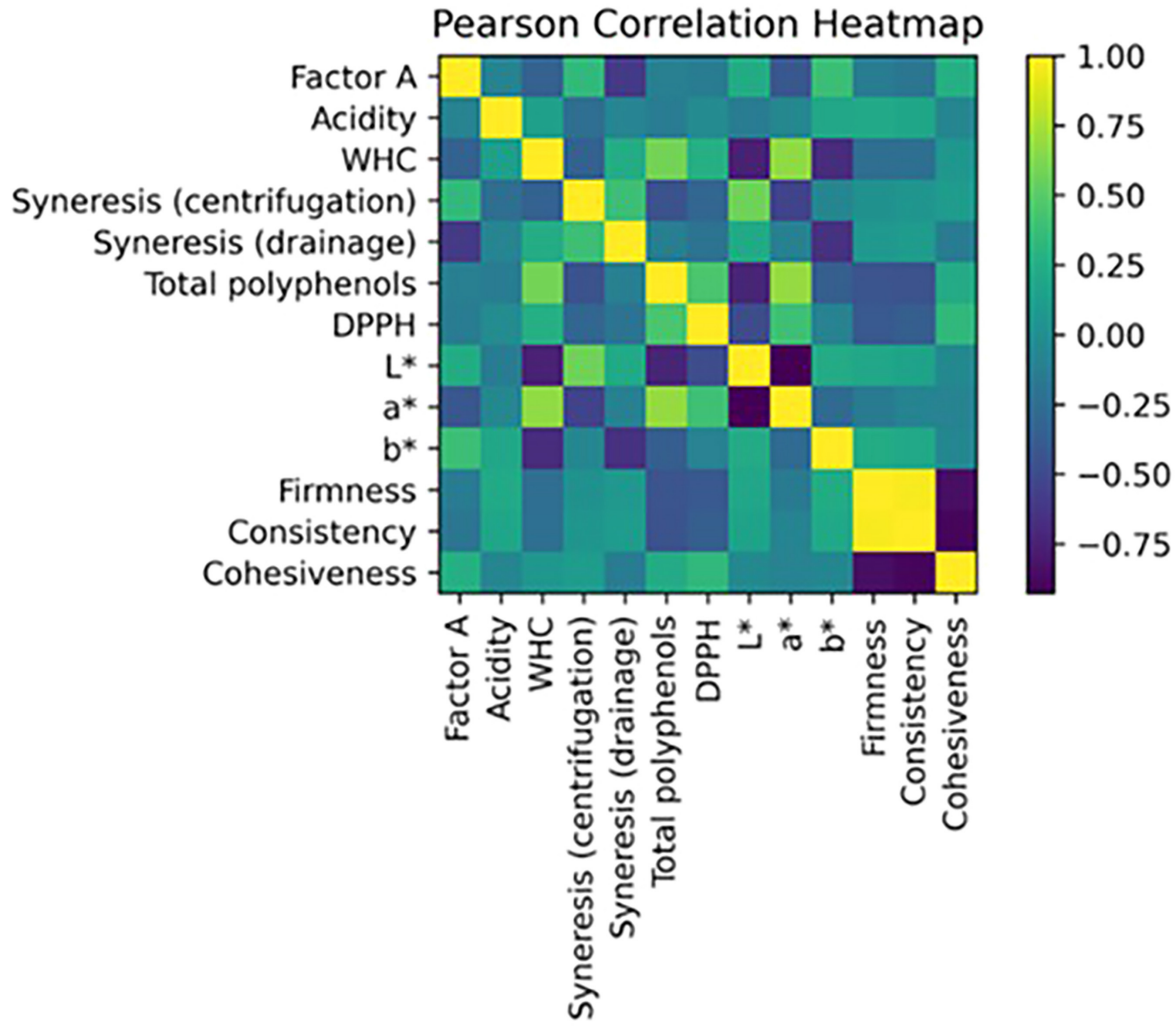
Pearson correlation heatmap.

In Figure 2, the Pearson correlation heatmap is presented. Several statistically significant correlations were identified between the analyzed parameters. A very strong positive correlation was observed between firmness and consistency (*r* = 0.9657**), indicating a close relationship between them. Strong negative correlations were found between consistency and cohesiveness (*r* = −0.8890**) and between firmness and cohesiveness (*r* = −0.8453**), indicating an inverse relationship between structural strength and cohesiveness. Color intensity reflects the strength and direction of the correlations. A very strong negative correlation was observed between L* and a* (*r* = −0.9257**), highlighting significant variations in color parameters. Water holding capacity (WHC) showed a strong negative correlation with L* (*r* = −0.7506**) and a moderate positive correlation with a* (*r* = 0.6753*). Total polyphenol content was negatively correlated with L* (*r* = −0.7263**) and positively correlated with a* (*r* = 0.6842*), indicating statistically significant associations between antioxidant compounds and color parameters. Overall, the correlation matrix highlights multiple significant relationships between physicochemical and textural parameters, indicating interdependence between structural, compositional, and functional properties of yogurt samples. These results indicate strong interactions between structural, color, and functional properties of yogurt, highlighting the complex relationships between formulation and quality attributes.

##### 3.2.3.2 Hierarchical cluster analysis (HCA)

**FIGURE 3:**
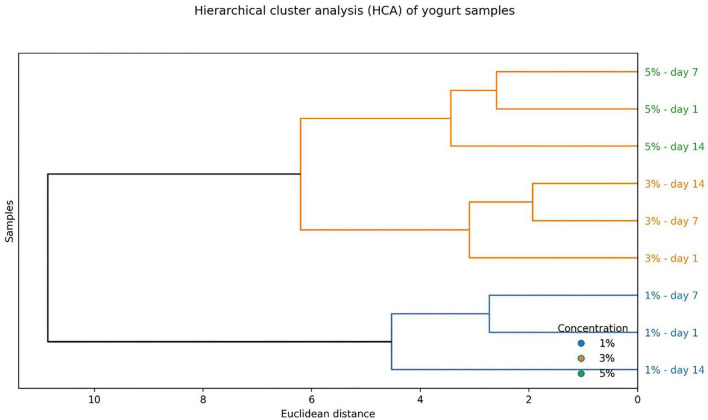
Hierarchical cluster analysis (HCA) of yogurt samples.

HCA was performed to evaluate the similarity between yogurt samples based on physicochemical, color, antioxidant, and textural parameters (Figure 3). The dendrogram revealed well-defined clustering patterns, indicating that the samples were not randomly distributed but grouped according to their compositional and structural characteristics. A clear separation of samples according to formulation (concentration) was observed. Samples with 1% concentration formed a distinct cluster, clearly separated from those with higher concentrations (3% and 5%), suggesting significant differences in their overall profiles. Within the clusters corresponding to higher concentrations, sub-clustering was observed, indicating similarities between samples with 3% and 5%, but still preserving distinctions between them. In addition, sub-clusters corresponding to storage time (1, 7, and 14 days) were identified within each concentration group, suggesting that storage time also influenced sample variability, although to a lesser extent than formulation. Overall, the cluster analysis confirms that formulation is the dominant factor influencing yogurt characteristics, while storage time contributes to secondary variations within each group.

## 4 Conclusion

The incorporation of cherry plum powder into yogurt significantly improves its antioxidant, rheological, and functional properties, making it a promising ingredient for the development of value-added dairy products. The results obtained varied depending on the powder concentration and storage period. Color parameters were only slightly affected by storage time. Lightness (L*) remained relatively stable, while a* and b* values showed minor variations, indicating limited changes in color during storage. Textural properties were influenced by storage time. Firmness increased in several samples, while consistency also showed an increasing trend (up to 4223 g⋅s). Cohesiveness exhibited slight variations, without a consistent trend across all samples. Total polyphenol content showed a marked increase with concentration, from 9.2 mg GAE/g at 1% to 19.0 mg GAE/g at 5%. Antioxidant activity also showed a slight increase, from 36.0% to 38.1%, suggesting improved functional properties. These findings highlight the potential of cherry plum powder and its effective incorporation into yogurt to improve nutritional, functional, and rheological properties. To the best of our knowledge, the use of this fruit in dairy products has not been thoroughly investigated to date. Therefore, for future research, we propose to continue this study by conducting antimicrobial and antioxidant analyses (using multiple methods), as well as developing other types of dairy products containing cherry plum powder. Future studies should include *in vivo* experiments to validate the health benefits and digestibility of yogurts enriched with cherry plum powder, further supporting their use in functional food markets.

## Data Availability

The original contributions presented in this study are included in this article/Supplementary material, further inquiries can be directed to the corresponding author.
