# Supplementary material for: Rheological profile and bioactive compounds content in yogurt incorporated with cherry plum fruit (Prunus Cerasifera Ehrh.) powders
Source: Front Nutr. 2026 Jul 30;13:1809167. doi: 10.3389/fnut.2026.1809167 (PMC13467823; doi:10.3389/fnut.2026.1809167)
Supplement: Supplementary file 1 [file Table_1.docx]

Supplementary Material

Rheological Profile and Bioactive Compounds Content in Yogurt Incorporated with Cherry Plum Fruit (*Prunus Cerasifera* Ehrh.) Powders

Ovidiu Tița^1^, Mădălina Moga^2^, Mihaela Adriana Tița^1^, Mihai Ognean^1^, Cecilia Georgescu^1^, Adina Frum^3^, Alina Constantina Florea^4^, Maria Adelina Constantinescu^1*^

*** Correspondence: Maria Adelina Constantinescu: adelina.constantinescu@ulbsibiu.ro**

# 1 Supplementary Tables

**Supplementary Table 4.** Textural, color, TPC and RSA characteristics of yogurt samples at different concentrations and storage times (mean ± standard deviation)

| **Concentration** | **Day** | **Firmness** | **Consistency** | **Cohesiveness** | **L*** | **a*** | **b*** | **TPC**  **mg/g d.w.** | **RSA**  **%** |
| --- | --- | --- | --- | --- | --- | --- | --- | --- | --- |
| 1% | 1 | 138.753 ± 16.322^a^ | 3610.443 ± 374.492^ab^ | 172.237 ± 19.243^a^ | 87.393 ± 1.712^a^ | 2.079 ± 1.109^b^ | 11.764 ± 2.287^b^ | 7.781 ± 1.845^c^ | 35.806 ± 0.887^ab^ |
| 3% | 1 | 125.332 ± 19.916^b^ | 3256.231 ± 320.491^b^ | 159.154 ± 19.028^a^ | 82.294 ± 3.297^b^ | 4.364 ± 2.376^a^ | 14.108 ± 3.993^a^ | 16.094 ± 3.594^b^ | 36.611 ± 0.848^a^ |
| 5% | 1 | 138.919 ± 11.655^a^ | 3687.622 ± 234.345^a^ | 179.692 ± 14.310^a^ | 78.036 ± 5.654^c^ | 5.522 ± 3.159^a^ | 14.297 ± 4.318^a^ | 19.700 ± 4.852^a^ | 37.708 ± 2.808^a^ |
| 1% | 7 | 131.264 ± 18.851^b^ | 3374.888 ± 467.576^b^ | 152.323 ± 20.427^b^ | 87.801 ± 1.725^a^ | 1.807 ± 0.866^c^ | 12.704 ± 1.827^b^ | 12.489 ± 5.876^b^ | 36.185 ± 1.323^b^ |
| 3% | 7 | 132.077 ± 35.740^b^ | 3322.445 ± 817.370^b^ | 150.002 ± 37.780^b^ | 80.693 ± 3.510^b^ | 4.463 ± 1.331^b^ | 14.968 ± 4.137^a^ | 18.615 ± 8.064^b^ | 37.761 ± 2.437^ab^ |
| 5% | 7 | 146.694 ± 27.621^a^ | 3679.181 ± 839.206^a^ | 167.288 ± 32.557^a^ | 76.320 ± 4.633^c^ | 5.921 ± 1.635^a^ | 16.297 ± 4.620^a^ | 21.491 ± 9.089^a^ | 39.010 ± 2.692^a^ |
| 1% | 14 | 169.925 ± 21.404^a^ | 4181.026 ± 337.323^a^ | 175.060 ± 17.324^a^ | 87.026 ± 2.469^a^ | 1.521 ± 1.067^c^ | 12.628 ± 2.308^b^ | 7.401 ± 3.611^c^ | 35.947 ± 2.296^b^ |
| 3% | 14 | 139.274 ± 39.839^b^ | 3544.441 ± 848.534^b^ | 149.660 ± 30.855^b^ | 80.148 ± 2.834^b^ | 4.494 ± 1.371^b^ | 14.707 ± 3.939^a^ | 13.057 ± 5.578^b^ | 37.115 ± 1.450^a^ |
| 5% | 14 | 164.346 ± 24.944^a^ | 4223.029 ± 563.214^a^ | 178.584 ± 17.636^a^ | 76.325 ± 3.716^c^ | 5.671 ± 1.747^a^ | 15.489 ± 4.441^a^ | 15.936 ± 5.580^a^ | 37.560 ± 1.896^a^ |

TPC: total polyphenol content; RSA: radical scavenging assay. The results are expressed as mean ± standard deviation (SD), n = 3. The results in the same column followed by the same letters are not significantly different (p < 0.05) according to Duncan’s multiple range test.

**Supplementary Table 5.** Influence of formulation (concentration) on color, antioxidant, polyphenols and textural properties of yogurt samples (mean ± standard deviation)

| **Concentration** | **Firmness** | **Consistency** | **Cohesiveness** | **L*** | **a*** | **b*** | **TPC**  **mg/g d.w.** | **RSA**  **%** |
| --- | --- | --- | --- | --- | --- | --- | --- | --- |
| 1% | 146.65 ± 20.04ᵇ | 3722.12 ± 420.37ᵇ | 166.54 ± 11.94ᵃ | 87.41 ± 0.39ᵃ | 1.80 ± 0.28ᶜ | 12.37 ± 0.49ᶜ | 9.22 ± 2.55ᶜ | 35.98 ± 0.19ᵇ |
| 3% | 132.23 ± 6.94ᶜ | 3374.37 ± 150.28ᶜ | 152.94 ± 4.78ᵇ | 80.38 ± 1.10ᵇ | 4.44 ± 0.07ᵇ | 14.59 ± 0.44ᵇ | 15.92 ± 2.78ᵇ | 37.16 ± 0.57ᵃᵇ |
| 5% | 149.99 ± 13.92ᵃ | 3863.28 ± 285.72ᵃ | 175.19 ± 6.31ᵃ | 76.89 ± 1.01ᶜ | 5.70 ± 0.20ᵃ | 15.36 ± 0.51ᵃ | 19.04 ± 2.80ᵃ | 38.09 ± 0.73ᵃ |

TPC: total polyphenol content; RSA: radical scavenging assay. The results are expressed as mean ± standard deviation (SD), n = 3. The results in the same column followed by the same letters are not significantly different (p < 0.05) according to Duncan’s multiple range test.

**Supplementary Table 6.** Pearson correlation coefficients (r) between physico-chemical and textural parameters of yogurt samples (*p < 0.05, p < 0.01)

|  | **Fact A** | **Acidity**  **°T** | **WHC** | **Syneresis through centrifugation**  **%** | **Syneresis through drainage\%** | **TPC**  **mg/g d.w.** | **RSA**  **%** | **L*** | **a*** | **b*** | **Firmness** | **Consistency** | **Cohesiveness** |
| --- | --- | --- | --- | --- | --- | --- | --- | --- | --- | --- | --- | --- | --- |
| **Fact A** | 1.0000 | -0.0904** | -0.3351 | 0.3401 | -0.6017* | -0.1125 | -0.1349 | 0.2423 | -0.4115 | 0.3866 | -0.1423 | -0.1871 | 0.2660 |
| **Acidity (°T)** | -0.0904** | 1.0000 | 0.1429 | -0.2346 | -0.0693 | -0.1232 | -0.0207 | -0.1333 | -0.0476 | 0.2007 | 0.2323 | 0.1944 | -0.0637 |
| **WHC** | -0.3351 | 0.1429 | 1.0000 | -0.3325 | 0.2513 | 0.5915 | 0.2637 | -0.7506** | 0.6753 | -0.6849 | -0.2367 | -0.2307 | 0.0734 |
| **Syneresis through centrifugation (%)** | 0.3401 | -0.2346 | -0.3325 | 1.0000 | 0.3930 | -0.4449 | -0.2991 | 0.5771 | -0.5350 | -0.0675 | 0.0361 | 0.0609 | 0.1146 |
| **Syneresis through drainage (%)** | -0.6017* | -0.0693 | 0.2513 | 0.3930 | 1.0000 | -0.1105 | -0.2025 | 0.2243 | -0.1051 | -0.6495 | 0.0774 | 0.1059 | -0.1321 |
| **TPC** (mg/g d.w.) | -0.1125 | -0.1232 | 0.5915 | -0.4449 | -0.1105 | 1.0000 | 0.4508 | -0.7263** | 0.6842 | -0.3608 | -0.4364 | -0.4467 | 0.2361 |
| **RSA (%)** | -0.1349 | -0.0207 | 0.2637 | -0.2991 | -0.2025 | 0.4508 | 1.0000 | -0.4804 | 0.3996 | -0.0839 | -0.4021 | -0.3611 | 0.3339 |
| **L*** | 0.2423 | -0.1333 | -0.7506** | 0.5771 | 0.2243 | -0.7263* | -0.4804 | 1.0000 | -0.9257** | 0.2343 | 0.1982 | 0.1655 | -0.0445 |
| **a*** | -0.4115 | -0.0476 | 0.6753 | -0.5350 | -0.1051 | 0.6842 | 0.3996 | -0.9257** | 1.0000 | -0.2665 | -0.1489 | -0.0780 | -0.0864 |
| **b*** | 0.3866 | 0.2007 | -0.6849 | -0.0675 | -0.6495 | -0.3608 | -0.0839 | 0.2343 | -0.2665 | 1.0000 | 0.2412 | 0.2116 | -0.0536 |
| **Firmness** | -0.1423 | 0.2323 | -0.2367 | 0.0361 | 0.0774 | -0.4364 | -0.4021 | 0.1982 | -0.1489 | 0.2412 | 1.0000 | 0.9657 | -0.8453 |
| **Consistency** | -0.1871 | 0.1944 | -0.2307 | 0.0609 | 0.1059 | -0.4467 | -0.3611 | 0.1655 | -0.0780 | 0.2116 | 0.9657 | 1.0000 | -0.8890 |
| **Cohesiveness** | 0.2660 | -0.0637 | 0.0734 | 0.1146 | -0.1321 | 0.2361 | 0.3339 | -0.0445 | -0.0864 | -0.0536 | -0.8453 | -0.8890 | 1.0000 |

WHC: water holding capacity; TPC: total polyphenol content; RSA: radical scavenging assay. The results indicate Pearson correlation coefficients (r) between the physicochemical and textural parameters of yogurt samples (*p < 0.05, p < 0.01).
